# Dr. Cayley, on Quackery

**Published:** 1800-11

**Authors:** Geo. Cayley

**Affiliations:** Durham


### *To the Editors of the Medical and Physical Journal.*

GENTLEMEN,

THROUGH the channel of your valuable Publication many important discoveries and improvements, in the practice of medicine and surgery, are daily communicated to the medical world, from which, without doubt, the public will derive the greatest benefit; and it must afford infinite satisfaction to every individual who contributes his mite towards the general fund of information, to find his endeavours thus powerfully seconded by so rapid and extensive a circulation as that of this work, which has been so long wanted.

To the old practitioner it will impart an increase of knowledge, to the young one it will give a *zest* for medical investigation; which, for want of a more general mode of communication than by private correspondence, was not sought after with the ardour its importance merits. Hence the utility of Medical Societies, where theories are brought face to face, and stand or fall as they are supported by by the vivid colours of truth, or wear the gloomy aſpect of hypothetical reaſoning. By the aid, however, of the Medical Journal, one general ſociety of the medical world is formed, affording to each member an opportunity of offering his opinion; which, to avoid giving offence to the whole, ought to be given with accuracy and candour, and judging others diſpaſſionately; medical controverſy, and warmth of temper, will not advance the ſcience, but will lower the individuals in the opinion of a diſcerning public.

Among the numerous advantages to be expected from this periodical work, is to be reckoned one, not of the leaſt importance; to wit, the extinction of prejudice amongſt perſons in higher ſituations of life, who are not of the faculty; for it is an incontrovertible truth, that new diſcoveries not unfrequently meet with more difficulties from the want of the ſanction of gentlemen of rank and fortune, than from the forbearance of medical authority. Thus, at this preſent moment, we meet with a powerful oppoſition to the general introduction of the Vaccine Pox, and to the application of Digitalis Purpurea in pulmonic affections: Does not the frequent fatality of the natural Small-pox, in this and every other country, juſtify an attempt to eradicate a diſorder that is deſervedly held in horror? And, however mild the inoculated Small-pox may be, it cannot, I think, afford any *favourable* argument againſt the Vaccine, which appears, almoſt beyond a doubt, to have exempted from the Variolous thoſe who have undergone the former; and, beſides, poſſeſſes ſo many advantages which the latter cannot boaſt of.

And do not the very few inſtances recorded of perſons having recovered from phthiſis pulmonalis, juſtify every rational attempt to defeat the opprobrium medici? I am much inclined to believe that, in the two firſt ſtages of the diſeaſe, the Fox-glove has produced wonderful effects, and will hope that by perſeverance and proper management, it may prove efficacious in the laſt ſtage.

A great deal has been ſaid on the ſubject of Quackery; I hope alſo to find a remedy againſt this evil in your very uſeful Journal.

In Ruſſia, where I was in regular practice during the ſpace of ſeven years, no one is allowed to viſit the ſick who has not been previouſly examined at the College of Phyſicians, under the following pains, viz. If he be a native, he is liable to be ſent into Siberia, and to have his property confiſcated; if a foreigner, to be ſent out of the empire. On the other hand, if any one be admitted by the College, notice is given through the medium of the St. Peterſburgh and Moſcow Gazettes, that the Faculty of Medicine find N. N. qualified to exercise his skill as Physician, Surgeon, or Accoucheur.

And when a medical gentleman writes a prescription, he is obliged to subjoin his name at full length; and the apothecaries (who dispense medicines only) in like manner add their own and the prescriber's name to the label, with the date of the prescription.

If corrosive sublimate, or any other deleterious drug, be prescribed, the medical gentleman is obliged himself to explain to the apothecary for what uses it is intended.

A few years ago the great importation of British quack medicines into Russia, caused the apothecaries to present a memorial to the College of Physicians, praying that the sale of them might be prohibited, and they were accordingly prohibited at St. Petersburgh; but, I believe, that in other places little attention was paid to the edict.

Although all the laws above mentioned were not put into execution, and though some of them, and others which I have not noticed, appear severe, yet, as a great national concern, I think some measures ought to be adopted in this country to prohibit Quackery, as it is an evil that is daily increasing amongst us, and it is not uncommon on the Continent to hear the English nation called "a country of Quacks and Dupes."

In the course of the ensuing month I shall transmit, for your approbation, some Medical Cases and Observations. I remain,

GENTLEMEN,

With great respect,

Your obedient servant,

*Durham,*
*Sept.* 29, 1800.

GEO. CAYLEY, M. D.

